# Orf-Virus-Infektion bei einer 53‑jährigen Frau

**DOI:** 10.1007/s00105-024-05412-w

**Published:** 2024-09-04

**Authors:** Katharina Anna Kälber, Alexander Enk, Janine Michel, Livia Schrick, Julia Katharina Winkler

**Affiliations:** 1 Universitäts-Hautklinik Heidelberg, Ruprecht-Karls Universität Heidelberg, Im Neuenheimer Feld 440, 69120 Heidelberg, Deutschland; 2 ZBS 1: Hochpathogene Viren & Konsiliarlabor für Pockenviren, Robert Koch-Institut, Berlin, Deutschland

**Keywords:** Parapocken, Lokalreaktion, Berufskrankheit, Therapie, Zoonosen, Parapoxvirus, Local reaction, Occupational illness, Treatment, Zoonoses

## Abstract

Das Orf-Virus gehört zu den Parapockenviren. Es kommt weltweit vor, sein natürliches Reservoir sind Schafe und Ziegen. Durch direkten oder indirekten Kontakt mit betroffenen Tieren kann es zu einer Infektion beim Menschen kommen. Es kommt zu infektiösen Ulzerationen, die bei immunkompetenten Patienten meist nach wenigen Wochen spontan abheilen. Eine Meldung an die zuständige Berufsgenossenschaft sollte erfolgen, sofern die Exposition im beruflichen Umfeld erfolgt, beispielsweise bei Bauern.

## Abstract (en)

The orf virus is one of the parapoxviruses. It occurs worldwide and its natural reservoir are sheep and goats. Infections in humans can occur through direct or indirect contact with affected animals. The infectious ulcerations usually heal spontaneously after a few weeks in immunocompetent patients. An infection must be reported to the responsible professional association if the exposure occurs in the occupational environment, for example in farmers.

## Anamnese

**Abb. 1:**
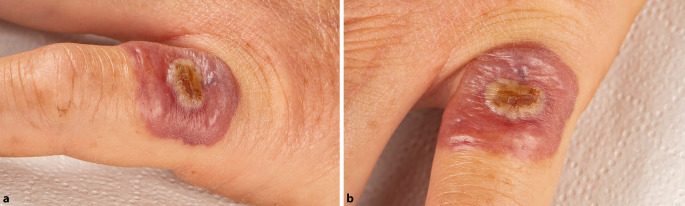
**a, b** Klinisches Bild: an der Streckseite des fünften Fingers der linken Hand ein ca. 2 cm messender blasserythematöser Nodus mit zentral krustös belegter Ulzeration

Wir berichten über den Fall einer 53-jährigen Frau, die sich notfallmäßig in unserer allgemeinen Ambulanz vorstellte mit einer neuen, schnell wachsenden Hautveränderung an der Streckseite ihres fünften Fingers der linken Hand (Abb. 1a). Anamnestisch war die Hautveränderung schmerzlos und nur leicht juckend. Die Patientin hatte keine Allgemeinsymptome, und es war keine Lymphadenopathie tastbar.

An Vorerkrankung war lediglich eine gut kontrollierte Hypothyreose bekannt, für die sie einmal täglich Levothyroxin 50 µg einnahm. Auf die Frage nach ihren Tätigkeiten gab sie an, mit Schafen und Ziegen zu arbeiten. Zudem berichtete die Patientin, dass einige Tiere in den letzten Wochen entzündete Wunden an Eutern oder um den Mund hatten.

## Untersuchung

Bei der klinischen Untersuchung zeigte sich ein erythematöser Nodus mit zentraler Ulzeration, der teilweise auch krustös belegt war (Abb. 1b).

## Diagnostik

Der von der Läsion der Patientin entnommene Abstrich zeigte sich in der Polymerasekettenreaktion positiv für Parapockenviren [1]. Durch eine weitere Typisierung gelang der Nachweis eines Orf-Virus (Sequenzierung von 595 Bp des „B2L-open reading frames“). So konnte die Verdachtsdiagnose einer Orf-Infektion (Ecthyma contagiosum) bestätigt werden.

## Therapie und Verlauf

Die Läsion unserer Patientin heilte innerhalb von wenigen Wochen unter einer antiseptischen Lokaltherapie vollständig ab. Anschließend besteht keine lebenslange Immunität und Reinfektionen sind möglich, welche jedoch rascher abheilen [2].

## Diskussion

Das *Orf-Virus* (ORFV) ist ein Vertreter der Parapockenviren, die komplex aufgebaut sind und eine doppelsträngige DNA besitzen. Weitere Vertreter sind unter anderem das Stomatitis-papulosa-Virus („bovine papular stomatitis virus“), Pseudokuhpockenvirus („pseudocowpox virus“) und das Seehundpockenvirus („sealpox virus“; [2]). Es handelt sich hierbei um Erreger von Zoonosen, die meist beruflich bedingt sind und klinisch nicht voneinander unterschieden werden können [3]. Das natürliche Reservoir von ORFV sind Schafe und Ziegen [4]. Es kommt weltweit vor und kann durch direkten oder indirekten Kontakt mit einem infizierten Tier oder Gegenständen auf den Menschen übertragen werden, auch einzelne Mensch-zu-Mensch-Übertragungen wurden beobachtet [5]. Eine jährliche Inzidenzspitze im Sommer, insbesondere in muslimischen Ländern, ist auf das jährliche islamische Opferfest Eid al-Adha zurückzuführen, bei dem die Tiere mit bloßen Händen geschlachtet und anschließend zubereitet werden [6]. ORFV ist sehr umweltresistent und konnte im Labor aus getrockneten Krusten nach mehreren Monaten bis Jahren wiedergewonnen werden [7]. Orf-Infektionen treten meist an den Handrücken oder an den Fingerstreckseiten auf, seltener ist in der Literatur ein Auftreten an ungewöhnlicheren Lokalisationen beschrieben, beispielsweise im Gesicht, genital, perianal und am Augenlid [8–10]. Bei immunkompetenten Patienten kommt es nach einer Inkubationszeit von 3–7 Tagen [4] meist zu einer singulären Läsion, die in 6 klinischen Stadien abläuft, die jeweils 7–14 Tage andauern (Tab. 1). Der Verlauf der Infektion dauert etwa 6–8 Wochen [11]. Insbesondere bei immunsupprimierten Patienten können auch atypische Verläufe mit ausgeprägten Ulzerationen auftreten [12, 13]. Es handelt sich um eine nicht meldepflichtige Erkrankung sowohl nach dem Infektionsschutz- als auch nach dem Tiergesundheitsgesetz, sodass keine verlässlichen Daten zur Prävalenz vorliegen. Unter den beruflich Exponierten scheint die Erkrankung zudem bekannt zu sein, sodass sie sich möglicherweise bei Symptomen der Erkrankung nicht ärztlich vorstellen, sondern eine Spontanheilung abwarten. Jedoch sollte bei Auftreten in einem beruflichen Zusammenhang eine Meldung an die zuständige Berufsgenossenschaft erfolgen. Manchmal kann es begleitend zu leichtem Fieber, Lymphadenopathie, Lymphangitis oder bakteriellen Superinfektionen kommen. In vereinzelten Fallberichten werden auch ein Erythema multiforme und ein Steven-Johnson-Syndrom als mögliche Komplikationen einer Orf-Infektion aufgeführt. Jedoch erhielten diese Patienten vorab systemisch Antibiotika, sodass hier ein kausaler Zusammenhang mit Orf-Infektionen nicht nachgewiesen werden kann [14, 15]. Lokale und systemische Antibiotikagaben sollten daher vermieden werden. Bei komplikationslosen Verläufen ist eine antiseptische Lokaltherapie ausreichend.

**Tab. 1:** Klinische Stadien der Orf-Infektion

Nr.	Stadium	Klinische Präsentation
1	Makulopapulöses Stadium	Es bilden sich einzelne oder mehrere, derbe ca. 2–3 cm große blasserythematöse bis violette Makulae und/oder Papeln
2	Kokardenstadium	Es entwickelt sich eine zentrale Rötung mit umgebendem weißem Ring und peripherer ein erythematöser Hof
3	Exsudativ seröses Stadium	Nun bildet sich eine nässende Oberfläche
4	Trockenes Stadium	Ausbildung einer Papel, die mit gelb-schwarzer Kruste bedeckt ist
5	Papillomatöses Stadium	Papillomatöse Umwandlung der Oberfläche
6	Regressionsstadium	Narbenlose Abheilung nach Abstoßung der Kruste

Impfungen sind derzeit in Deutschland für Tiere nicht verfügbar, ihre Wirksamkeit ist jedoch begrenzt. Für Menschen sind keine Impfstoffe verfügbar [16].

## Fazit für die Praxis

- Der Fall der hier vorgestellten Patientin verdeutlicht, wie wichtig eine spezifische Anamnese ist, um anschließend die zielführende Diagnostik einzuleiten und so zur richtigen Diagnose zu kommen.
- Der Nachweis eine Parapockenvirusinfektion erfolgt mithilfe von Hautabstrichen. Für das Labor ist relevant, dass die Verdachtsdiagnose explizit genannt wird, damit ggf. eine Weiterleitung der Probe an spezialisierte Labore erfolgen kann.
- Eine antiseptische Lokaltherapie ist in der Regel ausreichend, und Komplikationen, beispielsweise bakterielle Superinfektionen, sind selten.
- Bei beruflicher Exposition sollte eine Meldung an die zuständige Berufsgenossenschaft erfolgen.
